# The diagnostic accuracy of imaging modalities to detect pseudarthrosis after spinal fusion—a systematic review and meta-analysis of the literature

**DOI:** 10.1007/s00256-019-03181-5

**Published:** 2019-02-23

**Authors:** Marloes J. M. Peters, Carolien H. G. Bastiaenen, Boudewijn T. Brans, René E. Weijers, Paul C. Willems

**Affiliations:** 1 Department of Orthopaedic Surgery, Maastricht University Medical Center, Maastricht, The Netherlands; 2 Department of Epidemiology, Maastricht University, Maastricht, The Netherlands; 3 Department of Nuclear Medicine, University Hospital Ghent, Ghent, Belgium; 4 Department of Nuclear Medicine and Radiology, Maastricht University Medical Center, Maastricht, The Netherlands

**Keywords:** Meta-analysis, Spinal fusion, Diagnostics accuracy, Pseudarthrosis, Imaging

## Abstract

**Objective:**

The aim of the study was to determine the diagnostic accuracy of imaging modalities to detect pseudarthrosis after thoracolumbar spinal fusion, with surgical exploration as reference standard.

**Materials and methods:**

A systematic literature search for original studies was performed on the diagnostic accuracy of imaging as index test compared to surgical exploration as reference standard to diagnose pseudarthrosis after thoracolumbar spinal fusion. Diagnostic accuracy values were extracted and methodologic quality of studies was evaluated by the Quality Assessment of Diagnostic Accuracy Studies 2 (QUADAS-2) tool. Per modality, clinically comparable studies were included in subgroup meta-analysis and weighted odds ratios (ORs) were calculated using the random effects model.

**Results:**

Fifteen studies were included. Risk of bias was classified as high/unclear in 58% of the studies. Concerns of applicability was classified as high/unclear in 40% of the studies. Four scintigraphy studies including 93 patients in total were pooled to OR = 2.91 (95% confidence interval [CI]: 0.93–9.13). Five studies on plain radiography with 398 patients in total were pooled into OR = 7.07 (95% CI: 2.97–16.86). Two studies evaluating flexion-extension radiography of 75 patients in total were pooled into OR = 4.00 (95% CI: 0.15–105.96). Two studies of 68 patients in total were pooled for CT and yielded OR = 17.02 (95% CI: 6.42–45.10). A single study reporting on polytomography, OR = 10.15 (95% CI 5.49–18.78), was also considered to be an accurate study.

**Conclusions:**

With a pooled OR of 17.02, CT can be considered the most accurate imaging modality for the detection of pseudarthrosis after thoracolumbar spinal fusion from this review.

## Introduction

Low back pain is a global health and socio-economic problem [1], as it is the leading cause of disability and work absenteeism in the Western world [2]. When conservative measures fail, operative intervention can be considered. Spinal fusion is a surgical procedure in which rigid fixation of vertebral segments is achieved by means of osteosynthesis and bone grafting to create definite bony fusion of the vertebrae involved. Failed spinal fusion may occur in 30–40% of spinal fusion patients [3, 4]. Pseudarthrosis is defined as the absence of solid bony fusion at a minimum follow-up of 6 months after surgery [5, 6]. Pseudarthrosis can be associated with persistent or recurrent back and/or leg pain [7], but can also be asymptomatic [7–9]. Whether symptomatic or asymptomatic, pseudarthrosis increases the risk of material failure, late deformity, and neurological symptoms [10, 11].

Revision surgery is the preferred treatment in spinal fusion patients suffering from symptoms due to pseudarthrosis. Revision surgery is invasive, expensive, and may have a worse outcome than primary surgery [12, 13] and should only be performed when the pseudarthrosis diagnosis is irrefutable. Since symptoms of pseudarthrosis may be nonspecific and multiple individual sources of pain may contribute to the complex of symptoms [14], diagnostic tools are required to set the diagnosis. The gold standard for the diagnosis of pseudarthrosis is surgical exploration [5, 7, 15, 16], an invasive, costly, and nowadays rarely used test which is not desirable or ethical in patients without symptoms. The aim of the study was to determine the diagnostic accuracy of imaging modalities to detect pseudarthrosis after thoracolumbar spinal fusion, with surgical exploration as the reference standard.

## Materials and methods

### Identification of studies

This review was performed according to the PRISMA statement guidelines [17, 18]. A systematic literature search was conducted in the PubMed, EMBASE, and CINAHL databases from inception until February 2017 to identify relevant studies. A list of keywords and text words was formulated to describe the detection of pseudarthrosis by imaging as index test compared to surgical exploration as reference standard in patients after spinal fusion surgery. Terms for imaging: tomography, radiography, plain radiographs, MRI, CT, scintigraphy, SPECT, SPECT/CT, PET, PET/CT, DEXA. Terms for study design: diagnostic accuracy, precision, predictive value, sensitivity, specificity, false positive, false negative. Terms for patient population: spine, vertebrae, vertebral column, spinal fusion, spinal arthrodesis, spondylodesis, bone graft, pseudarthrosis, non-union, delayed union, clinical failure, surgical exploration, re-operation, second-look operation. The search was limited to the English language.

Once the search was completed, the resulting articles were checked for duplicates. Subsequently, two independent reviewers (PW, orthopedic surgeon with over 10 years of experience in spinal surgery and MP, junior researcher specialized in imaging) screened the identified citations to determine whether they met predefined in- and exclusion criteria. If disagreements could not be resolved by consensus, a third reviewer (CB, clinical epidemiologist with over 15 years of experience in conducting systematic reviews) was consulted. Only original studies that provided data to construct contingency tables were included. Exclusion criteria were spinal fusion for the indications bone fracture, tumor, infection; time interval between surgery and index test less than 6 months; patient population smaller than ten; cervical fusion; animal studies; in vitro studies.

### Data extraction

Standard reference data, population characteristics, details on spinal fusion, index test, reference test, and time intervals were extracted by the reviewers (PW, MP). Disagreements were resolved by consensus. Besides study characteristics, diagnostic accuracy data was extracted. Since the outcome was dichotomous (diagnosis was either pseudarthrosis or fusion), contingency tables were constructed. We also recorded whether the results originated from per-patient-, per-level-, or per-side-based analysis.

### Methodological quality

The methodological quality of each selected study was assessed independently by the reviewers according to the Quality Assessment for Diagnostic Accuracy Studies 2 (QUADAS-2) tool [19]. The QUADAS-2 tool consists of four key domains that discuss patient selection, index test, reference standard, flow of patients through the study, and timing of the index test and reference standard. Each domain was scored in terms of risk of bias and concerns regarding applicability to the research question. Disagreements were resolved by consensus.

### Data synthesis and statistical analysis

Pseudarthrosis was defined as a positive test result and fusion as a negative test result. Diagnostic accuracy values were calculated from the extracted contingency tables. Continuity correction was applied to studies with zero-cell counts by adding 0.5 to all cells of the study [20]. Per index test, the studies describing that test were considered for inclusion into subgroup meta-analysis.

### Inclusion in meta-analysis

Meta-analysis was only performed when studies evaluating the same modality were not significantly hampered by clinical heterogeneity. Studies were considered clinically heterogeneous when patient groups, outcome measures, and/or the execution of index tests were considerably different.

The random effect model was employed during meta-analyses to account for unobserved sources of variation [21]. The odds ratio (OR) was used as the principal summary measure in meta-analysis. The higher the OR, the better the discriminatory performance. An OR of 1 indicates a test that does not discriminate between patients with pseudarthrosis and patients with fusion [22]. An OR below 1 suggests a negative association between index test and surgical exploration. Analyses were performed using the Stata statistical software package, version 14.1 (StataCorp, College Station, TX, USA).

## Results

### Identification of studies

**Fig. 1:**
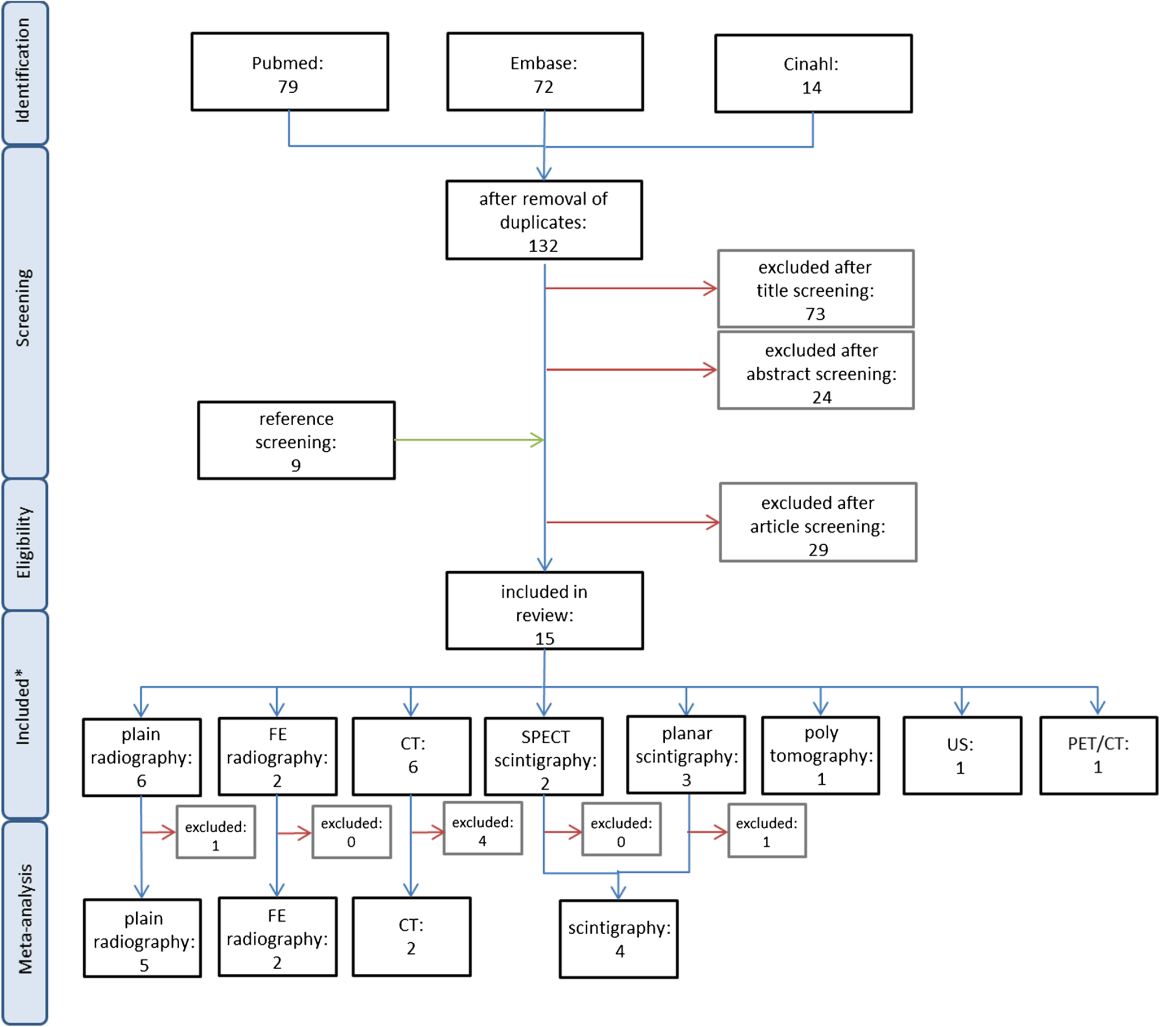
Flowchart showing the selection of studies from electronic search (identification) until inclusion in the subgroup meta-analyses. Initially, 165 potentially relevant references were identified through database search. One hundred thirty-two were obtained for further screening after removal of 33 duplicates. After removal based on title and abstract screening, the full text of 35 articles was screened and their reference sections were scanned for additional eligible studies. Hereafter, 15 studies were included this review, reporting on eight modalities. The meta-analysis part at the bottom of the figure will be discussed in ‘inclusion in meta-analysis’, which can be found hereafter in the result section. * 3 of the 15 studies described 2 to 4 modalities, leading to 22 included items

One hundred sixty-five potentially relevant references were identified through database search. After screening, 15 studies were included in this review, reporting on eight modalities: plain radiography, flexion extension radiography (FE radiography), computed tomography (CT), single-photon emission computed tomography (SPECT), planar scintigraphy, polytomography, ultra sound/sonography (US) and ^18^F-fluoride positron emission tomography/computed tomography (PET/CT). The study selection flowchart is detailed in Fig. 1. The level of evidence of the included studies ranged from I to III.

### Data extraction

Study characteristics of the 15 included studies are listed in Table 1. The number of levels fused in a single patient during initial surgery ranged from 1 to 13 levels. Eight articles monitored pseudarthrosis per patient, five monitored each level separately, and two made a distinction between the left and right side of each operated level. All articles reported that persistent low back pain and/or suspicion of pseudarthrosis was the reason for surgical exploration. The time interval between initial surgery and surgical exploration ranged from 6 to 120 months.

**Table 1:** Study characteristics

Author, year	Patient characteristics	Fusion surgery characteristics		Modality, index test			Surgical exploration (SE), reference standard		Time intervals	
		Indication	Fusion technique (number of patients/total)	Modality (number of patients/levels /sides)	Settings/protocol of modality	Definition pseudarthrosis (PA)	Description of intraoperative assessment	Definition pseudarthrosis (PA)	Between fusion surgery and modality	Between modality and SE
McMaster et al., 1980 [23]	- 110 patients in study and scored - average age: 13 years, age range: 8–25 years - multiple levels fused - SE for PA suspicion	Scoliosis	Instrumented interfacetal fusion (110/110), with posterior iliac crest autograft (86/110)	Scintigraphy (110 patients)	Scanning 3 h after intravenous injection of technetium-99 m; nuclear scanning gamma camera	Localized or generalized patchy areas of increased uptake	Removal of soft tissue; exploration of fusion mass; movement of spinous processes is noted	Irregular crevice filled with fibrous tissue; hairline PA; defective area in cancellous bone	6 months	2 days
Slizofski et al., 1987 [24]	- 26 patients in study, 11 patients were scored - median age: 58 years, age range: 27–71 years - 1–5 levels fused - SE for persistent low back pain	Degenerative disc or facet disease, spondylolisthesis	Bilateral transverse process fusion (16/26), posterior facet fusion (10/26); instrumented (19/26)	SPECT (11 patients)	Scanning 3 h after intravenous injection of technetium-99 m; 6-mm-thick tomograms	Focal areas of increased activity within the fusion mass noticed by ≥ 2 observers	NS	NS	Median: 20 months, range: 6–120 months	NS
Laasonen et al., 1988 [25]	- 48 patients in study, 20 patients were scored- age range: 16–64 years - 1–3 levels fused - SE for persistent low back pain w/or w/o radiating leg pain	Spondylolisthesis, chronic pain after disc surgery, miscellaneous	Posterolateral fusion (48/48) combined with intercorporal fusion (4/48)	CT (48 patients)	Slice thickness: 6 mm; reconstructions: selective sagittal	NS	Removal of fragmentation/ PA or widening of the spinal / nerve root canal	NS	7.3 years	NS
Brodsky et al., 1991 [15]	- 175 patients in study, 214 sides were scored - age range: 17–79 years - 1–6 levels fused - SE for hardware removal / PA suspicion / stenosis / radiculopathy	NS	Posterolateral fusion (175/175)	Plain radiography (214 sides)	Anteroposterior, lateral, oblique	NS	Visualization of bony bridging; manual manipulation	Lack of solid bony bridging; motion between vertebrae	Mean: 33.7 months	NS
				Polytomography (68 sides)	Anteroposterior, oblique	NS				
				FE radiography (64 sides)	Biplane bending films	NS				
				CT (42 sides)	NS	NS				
Blumenthal et al., 1993 [26]	- 49 patients in study and scored - age range: 22–54 years - 1–2 levels fused - SE for persistent low back discomfort	NS	Interbody fusion combined with posterolateral fusion (49/49)	Plain radiography (49 patients)	Anteroposterior, lateral	Blindly judged by two spinal surgeons and two musculoskeletal radiologists	Bone mass inspection; mechanical stress test (Kocher clamp)	No bridging bony mantle; motion	Average: 9 months	Just before surgery
Kant et al., 1995 [27]	- 75 patients in study, 126 levels were scored - age: NS - NS levels fused - SE for persistent low back pain / radiculopathy / PA on radiography / infection	NS	Posterolateral fusion (75/75), combined with interbody fusion with cancellous bone chips (37/75)	Plain radiography (126 levels)	Five views	No solid intertransverse bone or facet joint fusion, judged by an uninvolved orthopedic surgeon	Removal of hardware and soft tissue; exploration of motion, bone mass, facet joints, intertransverse process area	Absence of solid intertransverse bone mass or obliteration of facet joints; interspinous or facet motion	Mean: 51 weeks	1–4 weeks
Larsen et al., 1996 [14]	- 25 patients in study and scored - age: NS - 1–3 levels fused - SE for persistent and severe pain	NS	Instrumented posterolateral lumbar fusion (25/25)	Plain radiography (21 patients)	Anteroposterior, lateral, oblique; while standing	No bridging bony trabeculae	Removal of instrumentation; fusion inspection	NS	NS, fusion surgery to SE: > 1 year	NS
				FE radiography (11 patients)	While standing	> 3 degrees of motion				
				CT (24 patients)	Slice thickness: 5 mm; reconstructions: sagittal and coronal; overlapping slices	No bridging bony trabeculae				
				Scintigraphy (20 patients)	Intravenous injection of technetium-99 m	Increased uptake				
Albert et al., 1997 [28]	- 38 patients in study and scored - mean age: 42.8 years, age range: 22–73 years - NS levels fused - SE for persistent pain	Lumbar degenerative disc disease, kyphosis	Instrumented (35/38) spinal fusion (38/38)	SPECT (38 patients)	NS	Increased uptake beyond background signal judged by one blinded nuclear radiologist	Removal of instrumentation; subperiosteal inspection; stress testing using curettes	Motion between two fused levels	Average: 23.9 months, range: 9–120 months	NS
Jacobson et al., 1997 [29]	- 10 patients in study, 20 sides were scored - average age: 43.2 years, age range: 23–69 years - 1–13 levels fused - SE for persistent pain and PA suspicion	Postlaminectomy, scoliosis, spondylolisthesis	Instrumented (9/10) posterolateral fusion with iliac crest autograft (10/10)	Ultrasound (20 sides)	Patient in prone position; posterior approach; linear 7.5 MHz and curvilinear 5.0 MHz transducers	No bridging bone visible / presence of scattered and nonbridging echogenic foci at the fusion site, judged by one musculoskeletal radiologist	Removal of hardware; visual inspection of bridging bone; motion assessment	No solid bridging between vertebral segments	At least 9 months	Less than 1 week
Bohnsack et al., 1999 [30]	- 42 patients in study and scored - mean age: 42 years - average of 4 levels fused - SE for persistent back pain fusion area	NS	Dorsolateral fusion (32/42), combined procedures (10/42)	Scintigraphy (42 patients)	Intravenous injection of technetium-99 m	Report by radiologist of external institution	Removal of hardware	NS	Mean: 27 months	Just before SE
Brantigan et al., 2000 [31]	- 221 patients in study, 115 levels were scored - age range: 24–77 years - NS levels fused - SE for disabling back and/or radicular pain and degenerative change on MRI or discogram	Recurrent disc disease, spondylolisthesis, failed fusion	Instrumented posterior lumbar interbody fusion with cages (221/221)	Plain radiography (115 levels)	Standard	Lucency in bony bridging of the disc space	Fusion status examination	NS	NS, fusion surgery to SE: 24 months	NS
Carreon et al., 2007 [16]	- 93 patients in study, 163 levels scored - mean age: 57 years, age range: 19–86 years - 1–4 levels fused - SE for PA suspicion, painful instrumentation, adjacent level degeneration	NS	Instrumented posterolateral lumbar fusion (93/93)	CT (163 levels)	Slice thickness: 1 mm; reconstructions: sagittal and coronal	No obliteration of the facet joint space; interrupted trabeculated bone between transverse processes	Inspection of bony continuity; motion evaluation with laminar spreader between screwheads and pedicles	Noncontinuity of bone; presence of motion	NS, fusion surgery to SE average: 49 months	Average: 4 months
Fogel et al., 2007 [32]	- 90 patients in study, 172 levels were scored - average age: 43 years, age range: 27–70 years - NS levels fused - SE for persistent low back pain, adjacent level instability / stenosis / PA on radiography	Degenerative disc disease, failed back surgery and spondylolisthesis	Instrumented posterior lumbar interbody fusion with cages and iliac crest autograft, combined with posterolateral fusion (90/90)	Plain radiography (interbody, 172 levels)	Anteroposterior, lateral; parallel to each level	Two of the authors blindly and independently graded for evidence of interbody and posterolateral pseudarthrosis	Removal of hardware and soft tissue; exploration of fusion mass, facet joints, intertransverse areas; exploration of motion by distraction and compression	Posterolateral PA: defect in the bridging bone; visible motion in the posterolateral fusion area Interbody PA: any relative motion between segments	Before SE; fusion surgery to SE range: 12–65 months	NS
				Plain radiography (posterolateral, 172 levels)						
				CT (interbody, 109 levels)	Slice thickness: 1 mm; reconstructions: sagittal and coronal; high-speed helical CT scanner	Graded by 1 of 3 blinded radiologists; posterolateral PA = graft resorption; no fusion mass (Lenke B, C, D) interbody PA = collapse of construct, loss of disc height;cage displacement; bone resorption; graft lucency (BSF-1 and BSF-2)			Average: 30 months, Range: 10–60 months	NS
				CT (posterolateral, 109 levels)						
Carreon et al., 2008 [33]	- 49 patients in study, 69 levels scored - mean age: 43 years, age range: 21–65 years - 1–3 levels fused - SE for preoperative PA diagnosis / adjacent level degeneration	NS	Posteriorly instrumented (28/69) anterior interbody fusion with metallic cages (69/69)	CT (69 levels)	Slice thickness: fine-cut axial cuts reconstructions: sagittal and coronal; bone and soft tissue windows	Five experienced spine surgeons were asked to consider the disc space medial and lateral to cages, anterior and posterior to cages, and through cages	Inspection of fusion mass; distraction forces to detect motion	Absence of bony continuity; presence of motion across the fused levels	NS, fusion surgery to SE average: 22 months	NS
Quon et al., 2012 [34]	- 22 patients in study, 15 levels were scored - age range: 36–80 years - 1 level fused - SE for recurrent symptoms after spinal fusion, equivocal CT, based on PET/CT	NS	NS	^18^F-fluoride PET/CT (15 levels)	CT: slice thickness: 1.25–2 mm; 100–140 kV; 180–230 mAs; PET: scanning 45 min after intravenous injection of 222–370 MBq 18F-NaF	Nuclear medicine physician and a radiologist with musculoskeletal expertise reviewed the PET/CT images for lesions amenable to surgical intervention	Probing manually testing the fusion region for loosening and hardware failure at sites of abnormal tracer	NS	Range: 8–96 months	NS

*SE* surgical exploration, *PA* pseudarthrosis,* mm* millimeter, *NS* not specified, *MHz* megahertz, *kV* kilovolt, *mAs* milliampere second, *MBq* megabecquerel, *NaF* sodium fluoride

### Methodological quality assessment

**Fig. 2:**
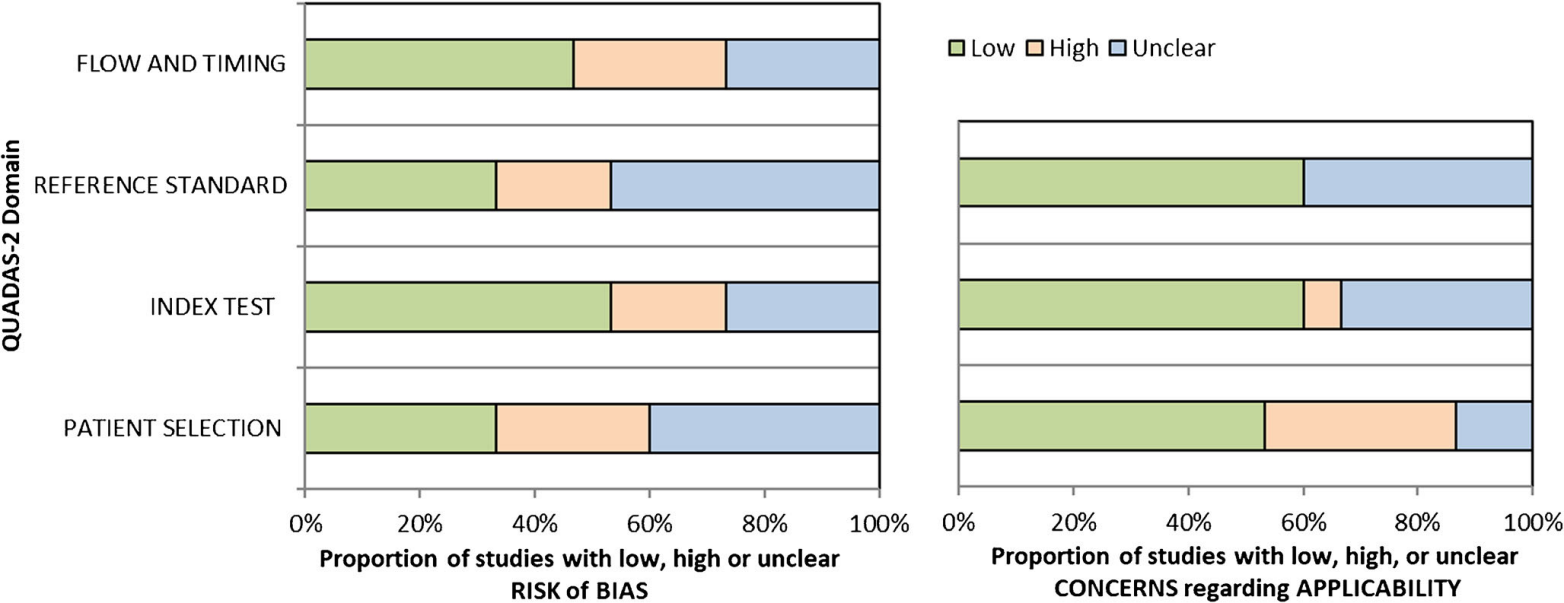
Stacked bar charts of QUADAS-2 scores presenting a quick overview of the methodological quality of the 15 included studies, expressed as a percentage of studies that met each criterion. For each quality domain, the proportion of included studies that suggest low, high, or unclear risk of bias and/or concerns regarding applicability are displayed in* green*,* orange*, and* blue*, respectively

Table 2 displays the quality assessment according to QUADAS-2. An overview of the distribution of QUADAS-2 scores is presented in Fig. 2. Risk of bias on ‘flow and timing’, ‘patient selection’, ‘index test’, and ‘reference standard’ was classified as high or unclear in 58% of cases. Common weaknesses related to poor documentation of patient selection and description of the reference standard. Two studies were considered to have low risk of bias in all four domains. Concerns of applicability on ‘patient selection’, ‘index test’, and ‘reference standard’ was classified as high or unclear in 42% of cases. Three studies were considered to suffer from low applicability concerns over all three domains.

**Table 2:**
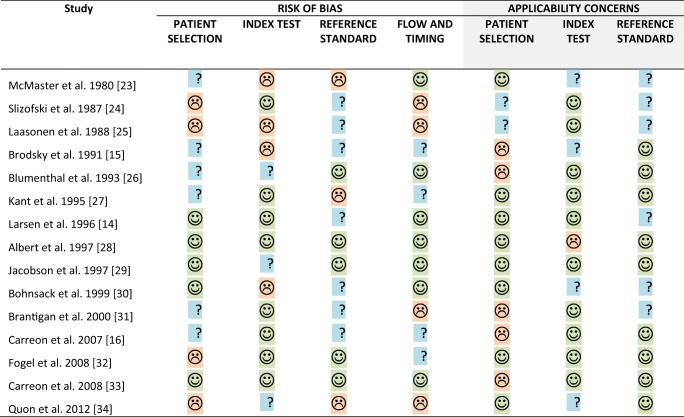
QUADAS-2 results for the 15 studies included in this review

### Data synthesis and statistical analysis

Table 3 shows the diagnostic accuracy values of the included studies, grouped per index test.

**Table 3:** Sensitivity, specificity, positive predictive value (PPV), negative predictive value (NPV), positive and negative likelihood ratios (LR+, LR-), prevalence of pseudarthrosis, accuracy ((true positive + true negative) / (total)) and OR values with corresponding 95% confidence intervals for the seven index tests

Author	Sensitivity	Specificity	PPV	NPV	LR+	LR-	Prevalence	Accuracy	OR	(95% CI)
Scintigraphy										
McMaster et al. 1980 [23]	0.86	0.94	0.50	0.99	14.71	0.15	0.06	0.94	97.00	(10.00–940.69)
Slizofski et al. 1987 [24]	0.75	0.83	0.94	0.50	4.50	0.30	0.77	0.77	15.00	(0.52–430.47)
Larsen et al. 1996 [14]	0.25	0.83	0.50	0.63	1.50	0.90	0.40	0.60	1.67	(0.18–15.13)
Albert et al. 1997 [28]	0.50	0.58	0.41	0.67	1.20	0.86	0.37	0.55	1.40	(0.37–5.27)
Bohnsack et al. 1999 [30]	0.50	0.92	0.40	0.95	6.33	0.54	0.10	0.88	11.67	(1.18–114.90)
Plain radiography										
Brodsky et al. 1991 [15]	0.60	0.89	0.78	0.76	5.30	0.45	0.41	0.77	11.77	(8.13–17.04)
Blumenthal et al. 1993 [26]	0.55	0.71	0.18	0.93	1.90	0.63	0.10	0.69	3.01	(1.55–5.84)
Kant et al. 1995 [27]	0.38	0.85	0.54	0.76	2.57	0.72	0.31	0.71	3.56	(1.48–8.52)
Larsen et al. 1996 [14]	0.89	0.42	0.53	0.83	1.52	0.27	0.43	0.62	5.71	(0.53–61.41)
Brantigan et al. 2000 [31]	0.55	0.97	0.67	0.95	18.91	0.47	0.10	0.93	40.40	(7.75–210.65)
Fogel et al. 2007 [32]	0.90	0.77	0.10	1.00	3.85	0.13	0.03	0.77	29.51	(1.55–560.03)
FE radiography										
Brodsky et al. 1991 [15]	0.37	0.96	0.86	0.71	9.74	0.66	0.39	0.73	14.86	(5.48–40.28)
Larsen et al. 1996 [14]	0.10	0.81	0.25	0.59	0.53	1.11	0.38	0.54	0.48	(0.02–14.70)
CT										
Laasonen et al. 1988 [25]	0.80	0.80	0.80	0.80	4.00	0.25	0.50	0.80	16.00	(1.78–143.15)
Brodsky et al. 1991 [15]	0.39	0.28	0.13	0.63	0.55	2.14	0.22	0.31	0.26	(0.12–0.57)
Larsen et al. 1996 [14]	0.78	0.53	0.50	0.80	1.67	0.42	0.38	0.63	4.00	(0.62–25.96)
Carreon et al. 2007 [16]	0.91	0.69	0.41	0.97	2.90	0.14	0.20	0.73	21.22	(6.11–73.67)
Fogel et al. 2007 [32]	0.90	0.70	0.13	0.99	3.03	0.14	0.05	0.71	21.29	(1.11–407.21)
Carreon et al. 2008 [33]	0.93	0.46	0.57	0.90	1.73	0.14	0.43	0.67	12.00	(2.51–57.48)
Polytomography										
Brodsky et al. 1991 [15]	0.84	0.65	0.73	0.79	2.44	0.24	0.53	0.75	10.15	(5.49–18.78)
US										
Jacobson et al. 1997 [29]	0.95	0.59	0.70	0.93	2.33	0.08	0.50	0.77	30.33	(1.39–660.76)
PET/CT										
Quon et al. 2012 [34]	0.97	0.25	0.91	0.50	1.29	0.13	0.88	0.88	9.67	(0.14–688.10)

### Inclusion in meta-analysis

The studies discussing the index tests SPECT [24, 28] and planar scintigraphy [14, 23, 30] were considered for inclusion into subgroup meta-analysis further referred to as scintigraphy. McMaster et al. was not included because the time interval between fusion surgery and surgical exploration was deviating too much from the other studies. The remaining four studies were pooled.

Six studies were considered for inclusion in meta-analysis for plain radiography [14, 15, 26, 27, 31, 32]. Fogel et al. was excluded since the low prevalence of pseudarthrosis made the study population incomparable to the other studies (see Table 3). The remaining five studies were considered comparable enough to be pooled. Two articles diagnosed pseudarthrosis per patient [14, 26], two per level [27, 31], and one per side [15]. We chose to pool these studies despite differences in analysis region since we were mainly interested in the correlation between findings on imaging and surgical exploration. Using the same rationale, no distinction was made between studies on posterolateral and interbody fusion.

Two articles were considered for FE radiography meta-analysis [14, 15]. Apart from differences in analysis regions, the study characteristics were considered comparable and the studies were therefore pooled.

Six articles were considered for inclusion in CT meta-analysis [14–16, 25, 32, 33]. The study of Brodsky et al. was excluded for lack of sagittal and coronal reconstructions, essential in the assessment of interbody bony fusion [14, 16, 33, 35]. Laasonen et al. and Larsen et al. were excluded on slice thickness. Thicknesses of 5 and 6 mm were used respectively, while bony bridging should be assessed using thin slice CT to be reliable [16, 32, 33, 35]. Fogel et al. was excluded for low prevalence of pseudarthrosis compared to the other studies. The posterolateral fusion patient group of Carreon et al. [16] and the interbody fusion patient group Carreon et al. [33] were pooled for CT.

**Fig. 3:**
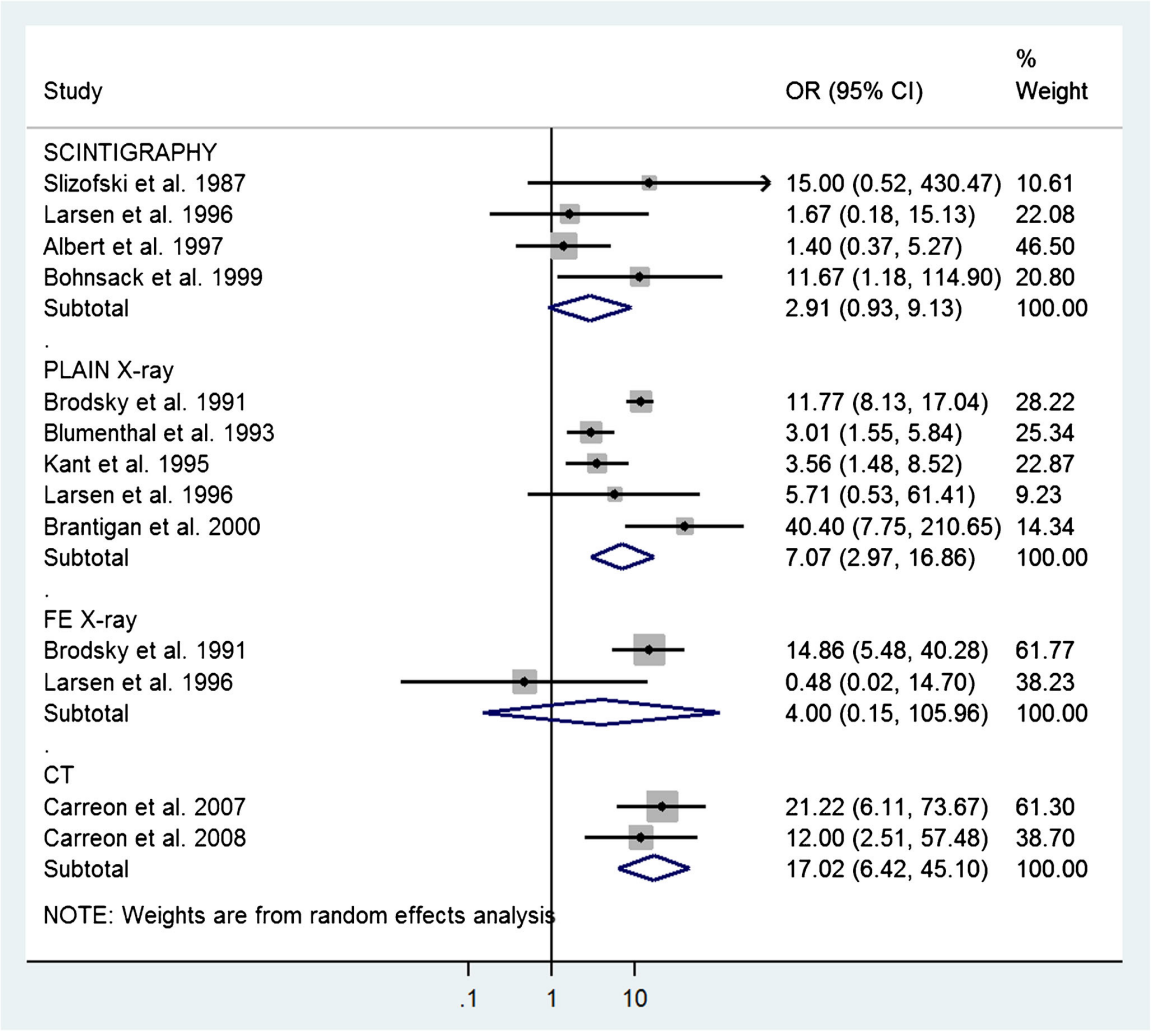
Forest plot of the included studies in the meta-analysis per modality. The size of each square is proportional to the study’s weight

Figure 3 shows a forest plot of the studies selected for subgroup meta-analysis, with their respective weights and resulting pooled ORs. Index tests for which only one study was identified, i.e., US, polytomography, ^18^F-fluoride PET/CT [15, 29, 34], could inevitably not undergo subgroup meta-analysis. These single studies were, however, evaluated on the same grounds and if considered reliable, included in Table 4 to complement the meta-analysis results. This was only the case for the study on polytomography [15]. For the study on US [29], the authors considered that with the evaluation of ten patients only, US was not investigated thoroughly enough for pseudarthrosis detection. In the ^18^F-fluoride PET/CT study [34], the reference standard was either surgical exploration or clinical follow-up, based on the index test outcome. This introduced a bias in the patient population that underwent surgical exploration; only patients with a suspicion of pseudarthrosis on ^18^F-fluoride PET/CT were surgically explored and used to calculate diagnostic accuracy.

**Table 4:** Overview of ORs as determined from included studies

	Number of studies	Number of patients	(Pooled) OR [95% CI]
Scintigraphy [14, 24, 28, 30]	4	93	2.91 [0.93–9.13]
Plain radiography [14, 15, 26, 27, 31]	5	398	7.07 [2.97–16.86]
FE radiography [14, 15]	2	75	4.00 [0.15–105.96]
CT [16, 33]	2	142	17.02 [6.42–45.10]
Polytomography [15]	1	68	10.15 [5.49–18.78]

## Discussion

This systematic review summarizes studies in literature that investigated the diagnostic accuracy of imaging modalities to detect pseudarthrosis after thoracolumbar spinal fusion with surgical exploration as the reference standard. Diagnostic accuracy values of individual studies were determined, and for studies of the same modality that were clinically comparable, a pooled OR was calculated.

Patients after spinal fusion can be monitored by several modalities. Plain radiographs attempt to reveal deficient morphology of the fusion mass as a sign of pseudarthrosis. However, plain radiographs are projections only [35, 36] whereas pseudarthrosis is a three-dimensional problem. The pooled OR of radiography was 7.07. In FE radiography, radiographs are made during flexion and extension of the spinal column to detect motion in the operated segment as a sign of pseudarthrosis. Cases exist where no signs of pseudarthrosis were found on plain radiography, CT, and MRI, but FE radiography detected the pseudarthrosis by unveiling motion between the segments [37]. However, on the other hand, absence of motion does not necessarily correspond with solid fusion and the presence of motion is not directly related to pseudarthrosis [12, 38–40]. Furthermore, no consensus exists on the threshold of allowable motion in a fused segment [40–42]. With a pooled OR of 4.00, FE radiography does not seem to outperform plain radiography. In polytomography, several radiographs along different sectional planes are taken. Going from a single slice in radiography to several planes in polytomography, the OR increased to 10.15. However, polytomography seems to be outdated by CT developments and currently not frequently used. CT offers three-dimensional osseous detail [33, 35]. After meta-analysis, CT was the modality with the highest OR in this review. Besides detection of bridging trabecular bone, CT is able to detect subsidence and lucency around fusion material as possible signs of pseudarthrosis [35]. On the downside, assessment can be complicated by artefacts when metallic cages and/or instrumentation are used [14, 32, 33, 35]. Technological improvements such as iterative reconstruction and dual-energy scanning are likely to improve accuracy [43]. Whether CT alone is sufficient for clinical decision-making is under debate. Choudhri et al. stated that multiple modalities should be considered for the noninvasive evaluation of symptomatic patients with suspected failure of spinal fusion [38]. US can demonstrate callus formation and bone healing [44, 45]. Although the first study assessing the role of US for pseudarthrosis detection in ten patients seemed promising in 1997 [29], it has been the only study since.

Pseudarthrosis diagnosis can also be based on abnormalities in bone metabolism. Studies on SPECT and planar scintigraphy were grouped together in meta-analysis since both modalities use ^99m^Tc-labeled phosphonates as tracer. ^99m^Tc-labeled phosphonates are adsorbed onto or into the crystalline structure of hydroxyapatite to mark bone remodeling. With a pooled OR of 2.91, scintigraphy amounted to the lowest OR value after subgroup meta-analyses. An analog to ^99m^Tc-labeled phosphonates is ^18^F-fluoride. Both tracers have similar uptake mechanisms [46] but ^18^F-fluoride decays via positron emission and can therefore be imaged by PET. Compared to ^99m^Tc SPECT, ^18^F-fluoride PET provides higher resolution, higher sensitivity, and better quantification capabilities [47]. PET combined with CT allows localization of abnormal uptake, which might enhance discriminative power [6]. Quon et al. evaluated PET/CT as index test for pseudarthrosis diagnosis [34]. The results seem promising but studies of higher methodological quality should be conducted to draw firmer conclusions on its value in pseudarthrosis diagnosis.

In the database search, one paper evaluating MRI [48] and one paper evaluating RSA as index test [49] were identified but not included. In MRI, bridging bone between endplates can be visualized [50] and changes in the vertebral body marrow signal as a sign of functional instability can be detected [48, 51]. On the downside, metal instrumentation complicates pseudarthrosis assessment in MRI. Length of follow-up was too short for the study of Lang et al. to be included. RSA is able to accurately quantify micromovements of vertebrae relative to each other, to evaluate lumbosacral stability [38, 42]. The study of Pape et al. could not be used to calculate the diagnostic accuracy of RSA for pseudarthrosis detection since all patients attained fusion.

A strength of the present review was that the patient populations of the included studies resemble patient populations that would undergo these tests in clinical practice to either confirm or exclude pseudarthrosis, since all suffered from persisting or recurrent pain after spinal fusion. The methodological choice to only include studies that compared an index modality to the gold standard of surgical exploration was a strength on one hand since it is the most valid way to assess the diagnostic accuracy of a modality [14]. However, it was a weakness on the other hand, since it meant the exclusion of newer studies that evaluate state-of-the-art modalities. The study design of using surgical exploration as gold standard is no longer ethical or practical in clinical practice. As a result, the value of state-of-the-art modalities could not be discussed in this review and are still left to be evaluated. Another weakness of the study was that studies in meta-analysis, although relatively comparable, did show differences in spinal fusion technique, types of cages and instrumentation, imaging characteristics, pseudarthrosis definition, experience of the observers, and patient characteristics. Especially the time interval between spinal fusion and index test was highly variable between studies. Furthermore, the interpretation of index test results was incomplete in some studies. Imaging findings were reported but not always classified as either pseudarthrosis or fused. In these cases, the cut-off point was determined by the writers of this review, which is arbitrary, although not necessarily far from clinical practice. Studies also reported poorly on patient population inclusion criteria. Lack of information may have led to incorrect inclusion of studies in meta-analyses and weakens the findings of this review.

To conclude, with a pooled OR of 17.02, CT can be considered the most accurate non-invasive imaging modality for the detection of pseudarthrosis after spinal fusion from this review.
